# Health Inequality Analysis in Europe: Exploring the Potential of the EQ-5D as Outcome

**DOI:** 10.3389/fpubh.2021.744405

**Published:** 2021-11-04

**Authors:** Inge Spronk, Juanita A. Haagsma, Erica I. Lubetkin, Suzanne Polinder, M. F. Janssen, G. J. Bonsel

**Affiliations:** ^1^Department of Public Health, Erasmus MC, University Medical Center Rotterdam, Rotterdam, Netherlands; ^2^Association of Dutch Burn Centers, Maasstad Hospital, Rotterdam, Netherlands; ^3^Department of Community Health and Social Medicine, The City University of New York School of Medicine, New York, NY, United States; ^4^Section Medical Psychology and Psychotherapy, Department of Psychiatry, Erasmus MC, Rotterdam, Netherlands; ^5^EuroQol Research Foundation, Rotterdam, Netherlands

**Keywords:** health inequality, Europe, EQ-5D, outcome, social determinants

## Abstract

**Objective:** This study explored the additive value of the multi-item EuroQol 5-Dimension 5-Level (EQ-5D-5L) as an outcome measure in health inequality analyses, relative to the single-item EuroQol visual analog scale (EQ VAS).

**Methods:** A sample comprising the general population from Italy, the Netherlands, and United Kingdom (UK) completed the EQ-5D-5L and the EQ VAS. The level of education was selected as a proxy for socio-economic status (SES). EQ-5D-5L level sum scores (LSS) were compared against EQ VAS scores. Stratified and multivariable analyses were used to study the associations between SES and the LSS/EQ VAS relative to the presence of chronic health conditions.

**Results:** A total of 10,172 people participated in this study. In the UK and Netherlands, the LSS was worst for respondents with a low educational level and better for respondents with middle and high educational levels. For Italy, the LSS was best for respondents with a middle educational level compared to respondents with low and high educational levels. The same patterns were observed for the EQ VAS, but differences were slightly smaller. Multivariable analyses showed generally stronger predictive relations in the UK, and with the LSS. The presence of chronic health conditions and being unable to work were independent strong predictors, canceling out the effects of education.

**Conclusions:** In three different European countries, the EQ-5D measures show the presence of education-dependent health inequalities, which are universally explained in regression analysis by independently the presence of chronic health conditions and the inability to work. In stratified analysis, the EQ-5D-5L LSS discriminates slightly better between participants with different levels of SES compared to the EQ VAS.

## Introduction

Health inequalities among population groups of various socioeconomic statuses (SES) are an important challenge for public health and social policy, both at the national and international levels (1). Existing health inequalities among countries largely have a socio-economic rather than a medical background (2, 3). Health inequalities are a societal concern; apart from the unfairness of the unequal share in good health *per se*, it is known that such inequalities impact economic and societal development (4).

Health inequality can be defined as the difference in health or health status between defined population groups (5). The uneven distribution of health determinants may be unjust or unfair and avoidable (5, 6). Regardless of the general level of development or prosperity, groups with a lower SES (measured by income, education, and/or occupation) have worse health outcomes, i.e., higher mortality rates and higher disease prevalence rates (7).

The difficulties of touching upon the causes and the resilience for change have resulted in a growing interest in the measurement and analysis of inequalities in health (8–10). So far, the focus has been mainly on the definition of pathways, the techniques to quantify inequalities, and the appropriate manner in which this topic can be communicated (1, 11, 12). Surprisingly, little attention has been paid to the health measure used as an indicator. In the analysis of clinical trials, health care performance measurement, and burden of disease research, the role of self-reported outcomes of patient/person on the health status has grown to the extent that this type of health information is regarded as critical (13–15). In health inequality analysis, however, simple dichotomous measures like mortality or disease indicators, and a single item 5- or 7-point subjective health scale are generally the norm (2, 7, 10, 16). Such simple measures facilitate the computation of inequalities, but also have some disadvantages. First, they give little information on the impact at the person level (general information only) and the contributing role of factors that require some specification on how they work. Second, the use of single-item self-report measures implies a smaller signal to noise ratio and a larger dependency on the homogeneous use of the response scale; consequently, the analytical power is then theoretically decreased (17–20).

Multi-item measures with a summary or utility outcome often outperform single-item measures (21). Therefore, these measures might be valuable in health inequality analyses as well. An example is the widely available and commonly used multidimensional health questionnaire, the EuroQol 5-Dimension (EQ-5D) (22). This self-report instrument is available in many languages, has been validated for many diseases and conditions, and has been used in health inequality assessment (23–26). The EQ-5D includes a descriptive system that consists of five dimensions, which include mobility, self-care, usual activities, pain/discomfort, and anxiety/depression, and the responses can be converted into a utility index or level sum score (LSS) (22, 27), thereby enabling burden of disease and cost-effectiveness estimations which are increasingly used for resource allocation and evaluation of care (28, 29). The EQ-5D also includes a visual analog scale (EQ VAS), which assesses a person's current (subjective) health status on a 0–100 scale. In the descriptive system of the EQ-5D, the extent of problems on each dimension is assessed, whereas in the EQ VAS, the extent of problems is translated into a score or rating of health status.

Theoretically, the multi-item EuroQol 5-Dimension 5-Level (EQ-5D-5L) might outperform the EQ VAS (or other single-item measures) in health inequality analyses, but this may be counterbalanced by the higher refinement of the EQ VAS scale (101 units). Therefore, the aim of this study was to explore the potential additive value of the EQ-5D-5L as an outcome measure in education-related health inequality analyses, relative to the EQ VAS, in a large three-country dataset (Italy, the Netherlands, and the UK). The secondary aim was to study the extent to which the EQ-5D-5L and EQ VAS scores are related to SES (in particular education) and to explore the role of chronic morbidity and other factors, which could play a mediating role.

## Materials and Methods

### Participants

During the period from 29th June to 31st July, 2017, a web-based survey was administered to members of the general public aged between 18 and 75 years, from three European countries (the UK, Italy, and the Netherlands) (30). Participants were recruited by Survey Sampling International. This market agency distributed and launched the surveys in the existing large internet panels. The samples were selected in such a way that they were representative of the population aged between 18 and 75 years in the countries with respect to age, gender, and educational level (Appendices 1A,B show the distribution of gender and age categories and educational level of the population in Italy, the Netherlands, and the UK). All the panel members had already provided informed consent to participate in online surveys. Informed consent for the present survey was obtained from all those who agreed to complete the questionnaire for this study. The present study was part of the CENTER-TBI study (EC grant 602150), and ethical approval was obtained from the Leids Universitair Centrum—-Commissie Medische Ethiek (approval P14.222/NV/nv). Only data from those respondents who completed the entire questionnaire were included in the analysis.

### Measures

The questionnaire covered socio-demographic information, including the country of residence (UK, Netherlands, or Italy), age, gender, highest achieved level of education, annual household income level, work status, and self-reported presence of chronic health conditions (e.g., asthma, chronic bronchitis, severe heart disease, consequences of a stroke, diabetes, severe back complaints, arthrosis, rheumatism, cancer, memory problems due to a neurological disease/dementia, memory problems due to aging, depression or anxiety disorder, and/or other chronic health conditions). One open field was available for recording the presence of any other chronic health conditions, which were categorized by one of the medically trained authors. Self-reported presence of chronic health conditions was recoded into two variables; one variable indicated if the respondent had chronic health conditions (“yes” / “no”) and another variable indicated the number of self-reported chronic health conditions. The level of education achieved was used as a proxy for SES, avoiding income parity issues between countries. Level of education was measured as the highest level achieved and coded based on the International Standard Classification of Education (ISCED-97) into three groups: up to lower secondary education (ISCED 0, 1, and 2; “low”), completed upper secondary education (ISCED 3 and 4; “mid”) and tertiary education (ISCED 5 and 6; “high”). Work status was categorized as employed (employee and self-employed), unemployed (consisting of those out of work for more than or less than 1 year), looking after others (e.g., a caregiver or parent), student, retired, and unable to work.

The questionnaire also included the EQ-5D-5L (27). The EQ-5D-5L includes five dimensions: mobility, self-care, usual activities, pain/discomfort, and anxiety/depression, with five ordered response categories, which include no problems, slight problems, moderate problems, severe problems, and extreme problems (27). Based on these five dimensions, a level sum score (LSS) can be calculated. The EQ-5D-5L LSS is defined as the equal-weight sum score of the five dimensions. It ranges from 5 (no problems on all dimensions: 1+1+1+1+1) to 25 (worst possible health state); thus, the LSS has 21 units. The EQ-5D-5L measure also includes a standardized visual analog scale (EQ VAS) for general health, which ranges from 0 (worst imaginable health) to 100 (best imaginable health) (31); hence, the scale has 101 units.

### Hypotheses

1) Compared to the EQ VAS, the EQ-5D-5L LSS is better able to discriminate between different educational levels, both in table analysis and regression analysis.
2) Respondents with a lower educational level have a higher (“worse”) EQ-5D-5L LSS and a lower EQ VAS score compared to the participants with a higher educational level.
3) When respondents are grouped based on the presence or absence of a chronic health condition, the group with chronic health conditions is expected to show less education-related health inequalities (neither EQ-5D-5L LSS nor EQ VAS), as it is assumed that education affects health perception stronger than while being diseased (32).
4) The health inequality effect, if present, was found to be the strongest in the UK, followed by the Netherlands and then Italy (7).

### Statistical Analysis

Descriptive statistics were used to assess respondent characteristics and health outcomes for the total sample and for the three countries separately. EQ-5D-5L LSS were transformed to a 0–100 scale in order to be comparable with the EQ VAS.

Transformed EQ-5D-5L level sum score (tLSS) = 100 − [(EQ-5D-5L level sum score − 5) x 5]

Transformed EQ-5D-5L level sum score and EQ VAS score were compared among the different countries, as well as between the different groups based on the level of education (low/middle/high) within each of the countries. The EQ-5D-5L tLSS was used as an analytical tool for assessing the overall combined performance of the five dimensions. This approach appeared very useful in an earlier study demonstrating the discriminatory power of EQ-5D when comparing different condition groups (33). Differences in mean EQ-5D-5L tLSS and EQ VAS scores were tested with ANOVA, and the differences in median scores were studied with the Kruskal–Wallis H-test. Subsequently, we compared the health outcomes within groups of respondents, with and without a specific health condition. The outcomes of the different groups, based on the level of education, were compared within each country and within these groups. Univariate analyses were used to test the relation between the respondent characteristics and the EQ-5D-5L tLSS, and between the respondent characteristics and the EQ VAS. Subsequently, multivariate stepwise regression analyses models (using backward elimination) were constructed, starting with sex and age in the first step. In the second step, the level of education, work status, income, and a number of chronic conditions were offered as potential explaining variables. This was done for each country separately. SPSS version 25 for Windows (IBM SPSS Statistics, SPSS Inc., Chicago, IL) was used for statistical analyses. Statistical significance was determined by a *p* < 0.05.

## Results

### Respondents

Total number of 10,172 respondents completed the survey. Of these, 3,026 respondents were from Italy, 3,027 were from the Netherlands, and 4,119 were from the UK. Characteristics of the respondents are presented in Table 1. The mean age of all the respondents was 44.5 years (SD 15.3), and the sample included an even representation of men and women. About half of the respondents had middle level education (49%), and a quarter of them had either low (26%) or high level education (25%), respectively. Most of the respondents were employed (52%). Household income was divided among the respondents as 22% (low), 41% (middle), 21% (high), and 16% (unknown). Half of the respondents had at least one chronic health condition. Depression or anxiety disorder was the most often reported chronic health condition (19%).

**Table 1:** Characteristics of the study population.

**Characteristic**	**Italy**	**Netherlands N (%)**	**UK N (%)**	**All countries N (%)**
	**(*n* = 3,026)**	**(*n* = 3,027)**	**(*n* = 4,119)**	**(*n* = 10,172)**
	**N (%)**	**N (%)**	**N (%)**	**N (%)**
**Sex**: Male	1,507 (49.8%)	1,520 (50.2%)	2,032 (49.3%)	5,059 (49.7%)
Age [Mean (SD)]	45.0 (14.8)	44.7 (15.3)	44.0 (15.6)	44.5 (15.3)
**Age/sex categories**				
**Male**				
18– <25 year	148 (4.9%)	188 (6.2%)	263 (6.4%)	599 (5.9%)
25– <40 year	384 (12.7%)	402 (13.3%)	602 (14.6%)	1,388 (13.6%)
40– <60 year	673 (22.2%)	619 (20.4%)	765 (18.6%)	2,057 (20.2%)
60–75 year	302 (10.0%)	311 (10.3%)	402 (9.8%)	1,015 (10.0%)
**Female**				
18– <25 year	142 (4.7%)	177 (5.8%)	271 (6.6%)	590 (5.8%)
25– <40 year	435 (14.4%)	412 (13.6%)	620 (15.1%)	1,467 (14.4%)
40– <60 year	644 (21.3%)	612 (20.2%)	777 (18.9%)	2,033 (20.0%)
60–75 year	298 (12.9%)	306 (10.1%)	419 (10.2%)	1,023 (10.1%)
**Level of education**				
Low	880 (29.1%)	811 (26.8%)	937 (22.7%)	2,628 (25.8%)
Middle	1,796 (59.4%)	1,420 (46.9%)	1,783 (43.3%)	4,999 (49.2%)
High	350 (11.6%)	796 (26.3%)	1,399 (34.0%)	2,545 (25.0%)
**Work status**				
Employed	1,494 (49.4%)	1,635 (54.0%)	2,176 (52.8%)	5,305 (52.2%)
Unemployed	704 (23.3%)	316 (10.4%)	365 (8.9%)	1,385 (13.6%)
Looking after others	120 (4.0%)	125 (4.1%)	277 (6.7%)	522 (5.1%)
Student	199 (6.6%)	209 (6.9%)	257 (6.2%)	665 (6.5%)
Retired	471 (15.6%)	386 (12.8%)	639 (15.5%)	1,496 (14.7%)
Unable to work	38 (1.3%)	356 (11.8%)	405 (9.8%)	799 (7.9%)
Household income^*^				
Low	871 (28.8%)	540 (17.8%)	870 (21.1%)	2,281 (22.4%)
Middle	1,146 (37.9%)	1,270 (42.0%)	1,732 (42.0%)	4,148 (40.8%)
High	510 (16.9%)	555 (18.3%)	1,070 (26.0%)	2,135 (21.0%)
Do not know/do not want to tell	499 (16.5%)	662 (21.9%)	447 (10.9%)	1,608 (15.8%)
^**^Self-reported chronic health conditions, overall prevalence/single prevalence				
Asthma, chronic bronchitis	7.3/3.2%	9.3/3.6%	12.9/4.8%	10.1/4.0%
Severe heart disease	2.4/0.9%	3.3/1.0%	3.0/0.8%	2.9/0.9%
Stroke (sequelae)	1.3/0.3%	2.3/0.8%	1.7/0.5%	1.7/0.5%
Diabetes	7.1/3.0%	7.6/2.5%	8.5/3.2%	7.8/2.9%
Severe back complaints and/or arthrosis	14.6/4.9%	17.5/6.5%	15.4/4.1%	15.8/5.1%
Rheumatism	6.7 /1.8%	6.1/1.2%	4.3/1.1%	5.6/1.3%
Cancer	1.9/0.9%	4.2/1.5%	2.8/1.0%	3.0/1.1%
Memory problems	5.7/1.4%	5.1/0.9%	6.3/1.1%	5.7/1.1%
Depression or anxiety disorder	14.5/7.1%	11.8/5.0%	26.9/11.9%	18.7/8.4%
Other chronic health condition	8.0/15.3%	16.5/25.6%	8.3/0.3%	10.7/7.6%
^***^Number of self-reported chronic health conditions				
No condition	1,683 (55.6%)	1,465 (48.4%)	1,921 (46.6%)	5,069 (49.8%)
1 condition	8,376 (27.7%)	936 (30.9%)	1,267 (30.8%)	3,040 (29.9%)
2 conditions	322 (10.6%)	370 (12.2%)	531 (12.9%)	1,223 (12.0%)
3 conditions	119 (3.9%)	150 (5.0%)	250 (6.1%)	519 (5.1%)
4 conditions	48 (1.6%)	72 (2.4%)	100 (2.4%)	220 (2.2%)
≥5 conditions	16 (0.5%)	34 (1.2%)	50 (1.2%)	101 (1.0%)

* *Income was grouped as low (the UK, <£14.000; Italy and the Netherlands, <€20.000), middle (UK, £14.000–£34.999; Italy, €20.000–€39.999; and the Netherlands, €20.000–€49.999), and high (the UK, more than £34.999; Italy, more than €39.999; and the Netherlands more than €49.999)*.

** *Prevalence of self-reported chronic health conditions, by type of health condition; overall prevalence: the sum of all patients reporting a specific health condition; single prevalence: the sum of patients only reporting that specific health condition, and thus having no other chronic health condition*.

*** *Total number of self-reported chronic health conditions*.

### Health Outcomes According to Country

**Figure 1:**
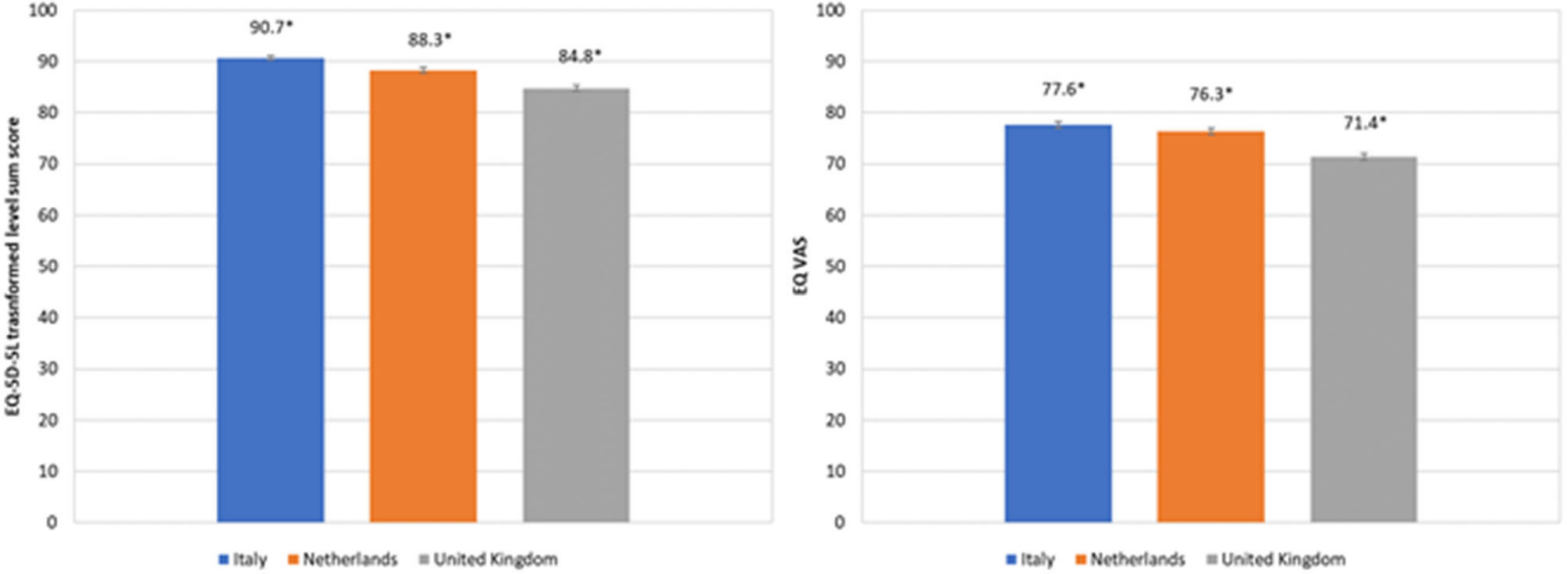
Mean (95% CI) EQ-5D-5L transformed level sum score and mean EQ VAS for the three different countries studied.

Mean outcomes of the health measures are shown in Figure 1, separately for each country. The tLSS was highest for respondents from Italy (mean: 90.7, SD: 11.8; median: 95.0, IQR: 85.0–100.0) and lowest for respondents from the UK (mean: 84.8, SD: 18.9; median: 90.0, IQR: 80.0–100.0). A similar pattern was seen for the EQ VAS; the EQ VAS score was the highest for respondents from Italy (mean: 77.6, SD: 17.4; median: 80.0, IQR: 70.0–90.0) and lowest for respondents from the UK (mean: 71.4, SD: 21.6; median: 78.0, IQR: 60.0–89.0).

### Health Outcomes According to the Level of Education

In Table 2, health outcomes are tabulated according to the level of education. In the UK and Netherlands, the tLSS was lowest (“worst”) for respondents with a low education level and invariably better for respondents with middle and high educational levels. For Italian respondents, a higher tLSS was observed for respondents with a middle educational level compared to their counterparts with a high educational level. For the EQ VAS, we observed the same pattern, but generally, differences in EQ VAS scores between respondents with low, middle, and high educational levels were slightly smaller compared to the tLSS.

**Table 2:** EQ-5D-5L transformed level sum score and EQ VAS outcomes for subgroups based on level of education in the three countries.

	**Italy**		**Netherlands**		**UK**	
	**Median (IQR)**	**Mean (SD)**	**Median (IQR)**	**Mean (SD)**	**Median (IQR)**	**Mean (SD)**
**EQ-5D-5L transformed level sum score**						
Education low	95.0 (85.0–100.0)	90.6 (12.0)^*^	90.0 (75.0–100.0)^*^	85.1 (16.3)^*^	90.0 (70.0–100.0)^*^	81.0 (21.0)^*^
Education middle	95.0 (85.0–100.0)	91.0 (11.4)^*^	95.0 (85.0–100.0)^*^	88.8 (14.1)^*^	90.0 (80.0–100.0)^*^	84.8 (19.1)^*^
Education high	95.0 (85.0–100.0)	89.2 (13.3)^*^	95.0 (85.0–100.0)^*^	90.6 (13.4)^*^	95.0 (80.0–100.0)^*^	87.4 (16.5)^*^
**EQ VAS**						
Education low	80.0 (70.0–90.0)	76.7 (18.8)	79.0 (61.0–90.0)^*^	74.0 (19.7)^*^	71.0 (50.0–87.0)^*^	67.2 (24.2)^*^
Education middle	80.0 (70.0–90.0)	78.2 (16.7)	80.0 (70.0–90.0)^*^	77.1 (17.5)^*^	78.0 (60.0–90.0)^*^	71.6 (21.7)^*^
Education high	80.0 (70.0–90.0)	77.0 (17.4)	80.0 (70.0–90.0)^*^	77.4 (17.4)^*^	80.0 (65.0–90.0)^*^	73.9 (19.0)^*^

* *Statistically significantly different between subgroups based on level of education; p < 0.05*.

### Health Outcomes for Subgroups With or Without a Chronic Health Condition

Mean and median tLSS and EQ VAS scores for subgroups of respondents with and without a particular chronic health condition are presented in Tables 3A,B. For each chronic health condition, statistically significant differences between respondents with low, middle, and high educational levels were observed for at least one outcome in at least one country, except for severe heart disease and rheumatism. Based on the tLSS, no statistically significant differences were found between subgroups based on the level of education in any country, among participants with severe heart disease, memory problems, and in those without chronic health conditions (Table 3A), whereas this was the case for participants with severe heart disease, stroke, rheumatism, and other chronic health conditions based on the EQ VAS (Table 3B). Overall, the number of statistically significant variables using the tLSS as the outcome was largely the same as when using EQ VAS.

**Table 3A:** EQ-5D-5L transformed level sum score per chronic health condition according to the level of education in the three countries.

**Chronic health condition**	**Italy**		**Netherlands**		**UK**	
**(number of participants with the condition)**	**Median (IQR)**	**Mean (SD)**	**Median (IQR)**	**Mean (SD)**	**Median (IQR)**	**Mean (SD)**
**Asthma, chronic bronchitis (*****n =*** **1,031)**	90.0 (80.0–95.0)	85.8 (14.6)	85.0 (70.0–95.0)	81.5 (17.3)	85.0 (65.0–95.0)	76.2 (23.3)
Education low (*n =* 282)	90.0 (80.0–96.3)	86.5 (15.6)	80.0 (70.0–95.0)^*^	77.7 (17.1)^*^	80.0 (50.0–92.5)^*^	70.6 (25.5)^*^
Education middle (*n =* 483)	90.0 (80.0–95.0)	85.9 (14.7)	85.0 (75.0–95.0)^*^	81.9 (16.3)^*^	85.0 (65.0–95.0)^*^	76.9 (23.2)^*^
Education high (*n =* 266)	85.0 (85.0–95.0)	84.7 (13.0)	95.0 (85.0–100.0)^*^	87.5 (18.5)^*^	90.0 (70.0–95.0)^*^	79.6 (21.0)^*^
**Severe heart disease (*****n =*** **295)**	90.0 (75.0–95.0)	83.6 (16.1)	80.0 (66.3–93.8)	78.5 (17.7)	75.0 (55.0–90.0)	68.7 (25.2)
Education low (*n =* 91)	95.0 (75.0–100.0)	85.4 (21.2)	80.0 (65.0–93.8)	78.3 (17.2)	65.0 (40.0–90.0)	62.6 (27.4)
Education middle (*n =* 132)	90.0 (75.0–95.0)	84.2 (13.7)	80.0 (68.8–90.0)	78.3 (16.8)	75.0 (51.3–90.0)	70.0 (24.0)
Education high (*n =* 72)	90.0 (70.0–92.3)	79.6 (18.9)	85.0 (68.8–95.0)	79.4 (21.5)	80.0 (55.0–90.0)	72.9 (24.0)
**Consequences of a stroke (*****n =*** **177)**	85.0 (72.5–91.3)	80.1 (17.9)	75.0 (60.0–90.0)	73.9 (19.2)	65.0 (53.8–86.3)	65.6 (23.2)
Education low (*n =* 57)	85.0 (55.0–90.0)	75.9 (18.4)	72.5 (56.3–90.0)	72.8 (19.9)	62.5 (53.8–86.3)^*^	63.8 (23.2)^*^
Education middle (*n =* 71)	90.0 (75.0–95.0)	81.8 (16.1)	80.0 (60.0–90.0)	75.3 (18.6)	70.0 (65.0–95.0)^*^	77.4 (20.0)^*^
Education high (*n =* 49)	90.0 (76.3–95.0)	81.9 (22.5)	75.0 (58.8–87.5)	72.8 (20.2)	60.0 (45.0–65.0)^*^	57.0 (22.4)^*^
**Diabetes (*****n =*** **797)**	90.0 (80.0–100.0)	86.3 (15.7)	85.0 (65.0–95.0)	79.0 (18.4)	80.0 (55.0–95.0)	73.5 (23.2)
Education low (*n =* 280)	95.0 (85.0–100.0)	89.8 (11.8)	75.0 (60.0–95.0)^*^	74.3 (19.3)^*^	75.0 (55.0–90.0)	70.6 (24.7)
Education middle (*n =* 342)	90.0 (80.0–100.0)	85.5 (15.6)	85.0 (70.0–95.0)^*^	82.2 (16.9)^*^	77.5 (60.0–95.0)	74.6 (22.9)
Education high (*n =* 175)	90.0 (76.3–98.8)	82.3 (21.1)	87.5 (70.0–100.0)^*^	82.8 (17.5)^*^	80.0 (62.5–95.0)	75.5 (21.4)
**Severe back complaints and/or arthrosis (*****n =*** **1,605)**	85.0 (70.0–90.0)	79.3 (15.3)	80.0 (65.0–90.0)	75.4 (16.9)	65.0 (45.0–80.0)	63.3 (22.8)
Education low (*n =* 534)	85.0 (70.0–90.0)	79.6 (16.0)	75.0 (65.0–85.0)^*^	73.6 (16.4)	60.0 (45.0–80.0)^*^	59.9 (4.6)^*^
Education middle (*n =* 746)	85.0 (70.0–90.0)	79.2 (14.4)	75.0 (65.0–90.0)^*^	75.7 (17.0)	70.0 (45.0–80.0)^*^	62.6 (4.6)^*^
Education high (*n =* 325)	80.0 (70.0–90.0)	79.0 (17.5)	80.0 (70.0–90.0)^*^	78.3 (17.5)	75.0 (55.0–85.0)^*^	68.7 (4.3)^*^
**Rheumatism (*****n =*** **568)**	85.0 (75.0–90.0)	80.1 (16.5)	70.0 (60.0–85.0)	71.0 (16.5)	75.0 (50.0–85.0)	66.9 (23.4)
Education low (*n =* 204)	85.0 (70.0–95.0)	79.5 (17.9)^*^	80.0 (70.0–95.0)	69.7 (14.5)	72.5 (50.0–85.0)	65.7 (22.5)
Education middle (*n =* 265)	85.0 (78.8–90.0)	82.2 (14.2)^*^	85.0 (75.0–95.0)	72.3 (17.0)	75.0 (47.5–90.0)	66.6 (24.9)
Education high (*n =* 99)	75.0 (70.0–90.0)	73.0 (20.7)^*^	95.0 (85.0–100.0)	70.8 (19.8)	80.0 (52.5–85.0)	69.4 (22.8)
**Cancer (*****n =*** **301)**	85.0 (75.0–95.0)	81.9 (17.5)	85.0 (70.0–95.0)	81.4 (17.3)	85.0 (65.0–95.0)	77.7 (22.3)
Education low (*n =* 80)	90.0 (77.5–95.0)	82.3 (20.8)	75.0 (65.0–95.0)^*^	76.1 (20.3)^*^	85.0 (45.0–90.0)	70.8 (25.5)
Education middle (*n =* 141)	85.0 (75.0–95.0)	82.4 (16.0)	85.0 (70.0–95.0)^*^	80.9 (16.5)^*^	85.0 (70.0–95.0)	82.3 (15.2)
Education high (*n =* 80)	85.0 (75.0–95.0)	78.6 (21.0)	95.0 (80.0–100.0)^*^	88.3 (12.3)^*^	90.0 (65.0–100.0)	77.7 (25.4)
**Memory problems (*****n =*** **584)**	87.5 (75.0–95.0)	82.7 (15.5)	75.0 (60.0–90.0)	74.6 (18.2)	70.0 (45.0–85.0)	65.5 (24.2)
Education low (*n =* 187)	90.0 (80.0–92.5)	84.2 (14.1)	75.0 (60.0–90.0)	73.6 (19.3)	65.0 (45.0–80.0)	63.2 (22.2)
Education middle (*n =* 264)	90.0 (75.0–95.0)	82.2 (16.1)	75.0 (60.0–90.0)	75.1 (17.0)	75.0 (42.5–90.0)	65.7 (26.8)
Education high (*n =* 133)	85.0 (75.0–95.0)	82.0 (16.4)	80.0 (62.5–92.5)	75.8 (19.7)	70.0 (50.0–90.0)	67.6 (22.5)
**Depression or anxiety disorder (*****n =*** **1,903)**	85.0 (75.0–90.0)	80.2 (13.7)	80.0 (65.0–90.0)	76.3 (16.1)	75.0 (60.0–90.0)	70.2 (21.5)
Education low (*n =* 497)	85.0 (75.0–90.0)	81.3 (12.7)	75.0 (60.0–85.0)^*^	72.6 (17.2)^*^	70.0 (50.0–85.0)^*^	64.3 (22.5)^*^
Education middle (*n =* 918)	85.0 (75.0–90.0)	79.9 (14.1)	80.0 (70.0–90.0)^*^	76.8 (16.0)^*^	75.0 (55.0–90.0)^*^	69.8 (22.0)^*^
Education high (*n =* 488)	85.0 (75.0–90.0)	79.4 (13.6)	85.0 (75.0–90.0)^*^	81.2 (13.0)^*^	80.0 (65.0–90.0)^*^	74.9 (18.7)^*^
**Other chronic health conditions (*****n =*** **1,084)**	90.0 (80.0–95.0)	84.4 (14.5)	85.0 (70.0–95.0)	79.6 (16.6)	70.0 (50.0–85.0)	66.6 (23.1)
Education low (*n =* 313)	85.0 (67.5–95.0)	81.0 (16.0)^*^	80.0 (65.0–90.0)^*^	76.1 (17.6)^*^	70.0 (45.0–85.0)^*^	64.6 (25.0)^*^
Education middle (*n =* 518)	90.0 (80.0–95.0)	85.8 (13.1)^*^	85.0 (70.0–95.0)^*^	80.2 (16.3)^*^	65.0 (45.0–80.0)^*^	63.4 (23.0)^*^
Education high (*n =* 253)	90.0 (80.0–95.0)	86.5 (15.4)^*^	85.0 (75.0–95.0)^*^	83.1 (14.9)^*^	75.0 (60.0–90.0)^*^	72.1 (20.7)^*^
**No chronic health conditions (*****n =*** **5,046)**	100.0 (95.0–100.0)	95.6 (6.7)	100.0 (95.0–100.0)	95.9 (7.6)	100.0 (95.0–100.0)	95.3 (8.6)
Education low (*n =* 1,160)	100.0 (95.0–100.0)	95.3 (7.3)	100.0 (95.0–100.0)	95.5 (8.4)	100.0 (95.0–100.0)	94.8 (9.5)
Education middle (*n =* 2,583)	100.0 (95.0–100.0)	95.7 (6.3)	100.0 (95.0–100.0)	96.0 (6.7)	100.0 (95.0–100.0)	95.1 (8.3)
Education high (*n =* 1,326)	100.0 (90.0–100.0)	95.5 (6.8)	100.0 (95.0–100.0)	96.0 (8.2)	100.0 (95.0–100.0)	95.6 (8.5)

* *Statistically significantly different between subgroups based on level of education; p < 0.05*.

**Table 3B:** EQ VAS per chronic health condition according to the level of education in the three countries.

**Chronic health condition**	**Italy**		**Netherlands**		**UK**	
**(number of participants with the condition)**	**Median (IQR)**	**Mean (SD)**	**Median (IQR)**	**Mean (SD)**	**Median (IQR)**	**Mean (SD)**
**Asthma, chronic bronchitis (*****n =*** **1,031)**	79.0 (60.0–87.0)	72.0 (19.8)	70.0 (60.0–82.5)	68.8 (20.0)	70.0 (49.8–81.0)	63.8 (23.1)
Education low (*n =* 282)	79.0 (50.0–90.0)	68.6 (23.8)	65.0 (51.0–80.0)^*^	64.7 (21.0)^*^	60.0 (40.0–80.0)^*^	59.1 (25.0)^*^
Education middle (*n =* 483)	80.0 (60.0–87.0)	72.6 (19.2)	70.0 (60.0–81.0)^*^	68.4 (19.6)^*^	70.0 (50.0–81.0)^*^	65.1 (23.3)^*^
Education high (*n =* 266)	77.0 (66.0–85.0)	74.6 (14.7)	80.0 (70.0–90.0)^*^	77.0 (16.8)^*^	70.0 (50.8–81.0)^*^	65.9 (21.0)^*^
**Severe heart disease (*****n =*** **295)**	70.0 (50.0–80.0)	64.7 (20.4)	70.0 (51.0–79.8)	64.7 (19.9)	60.0 (40.0–80.0)	57.0 (25.7)
Education low (*n =* 91)	76.0 (45.0–80.0)	64.9 (30.3)	70.0 (60.0–76.5)	66.2 (15.5)	50.0 (31.0–74.5)	50.6 (25.8)
Education middle (*n =* 132)	67.5 (50.0–79.3)	64.3 (17.1)	64.0 (50.0–82.0)	63.5 (22.9)	60.5 (43.8–79.0)	59.3 (23.3)
Education high (*n =* 72)	69.0 (46.0–82.5)	65.8 (21.1)	70.0 (50.0–78.8)	64.3 (21.6)	69.0 (40.0–80.0)	60.6 (27.7)
**Consequences of a stroke (*****n =*** **177)**	76.0 (50.8–86.8)	66.5 (25.3)	63.0 (55.0–79.5)	64.8 (18.5)	62.0 (35.0–80.3)	58.5 (27.9)
Education low (*n =* 57)	69.0 (59.0–82.0)	65.5 (24.3)	61.0 (59.0–80.8)	65.2 (21.7)	50.0 (31.0–79.5)	53.4 (31.1)
Education middle (*n =* 71)	80.0 (50.0–90.0)	66.8 (26.7)	64.0 (51.0–80.0)	65.8 (16.0)	74.0 (60.5–80.0)	69.0 (19.4)
Education high (*n =* 49)	78.0 (43.5–89.0)	67.0 (26.5)	64.0 (55.5–71.3)	62.6 (19.7)	45.0 (33.0–83.0)	54.6 (29.2)
**Diabetes (*****n =*** **797)**	75.0 (61.0–85.0)	71.9 (18.0)	70.0 (54.0–80.0)	67.2 (19.3)	68.0 (41.0–80.0)	61.1 (24.1)
Education low (*n =* 280)	77.0 (62.0–90.0)	73.6 (18.6)	62.0 (50.0–80.0)^*^	63.7 (20.9)^*^	60.0 (40.0–80.0)	58.3 (24.3)
Education middle (*n =* 342)	75.0 (61.0–84.0)	72.0 (17.0)	70.0 (56.5–81.8)^*^	67.6 (19.5)^*^	64.0 (47.5–80.0)	61.9 (23.6)
Education high (*n =* 175)	72.5 (58.0–80.5)	67.9 (20.7)	75.0 (60.8–81.3)^*^	73.3 (13.6)^*^	70.0 (44.0–81.5)	63.6 (24.4)
**Severe back complaints and/or arthrosis (*****n =*** **1,605)**	70.0 (52.0–81.0)	67.0 (19.7)	70.0 (56.0–80.0)	66.6 (19.6)	59.0 (40.0–71.3)	54.7 (23.3)
Education low (*n =* 534)	70.0 (51.0–81.0)	67.2 (20.8)	70.0 (51.0–80.0)	64.3 (19.5)	50.0 (38.3–70.0)^*^	50.4 (23.0)^*^
Education middle (*n =* 746)	70.0 (55.0–80.0)	67.1 (19.0)	70.0 (57.0–80.0)	67.1 (19.8)	60.0 (33.5–72.3)^*^	54.7 (23.4)^*^
Education high (*n =* 325)	67.0 (50.0–81.0)	65.9 (19.7)	70.0 (60.0–84.3)	69.5 (18.9)	62.5 (50.0–75.0)^*^	60.1 (22.4)^*^
**Rheumatism (*****n =*** **568)**	71.0 (59.3–81.0)	68.6 (20.4)	60.0 (50.0–74.3)	60.4 (19.0)	60.0 (40.0–77.5)	56.6 (23.9)
Education low (*n =* 204)	72.0 (59.0–85.0)	68.5 (24.2)	60.0 (46.5–70.5)	59.0 (18.6)	60.0 (40.0–72.8)	56.2 (22.7)
Education middle (*n =* 265)	73.5 (60.0–81.0)	70.0 (18.7)	61.0 (50.0–79.0)	61.6 (18.5)	60.0 (38.5–80.0)	56.4 (24.5)
Education high (*n =* 99)	61.0 (51.0–75.0)	63.3 (17.2)	60.0 (50.0–75.0)	60.8 (21.2)	61.0 (40.0–80.0)	57.5 (25.4)
**Cancer (*****n =*** **301)**	70.0 (50.0–82.0)	67.0 (20.1)	71.0 (60.0–86.0)	70.4 (20.2)	69.5 (45.3–81.0)	61.8 (24.3)
Education low (*n =* 80)	75.0 (50.0–81.5)	65.4 (23.3)	70.0 (57.5–85.5)	68.5 (21.0)	50.5 (31.0–76.0)^*^	52.5 (25.4)
Education middle (*n =* 141)	70.0 (50.0–82.0)	67.9 (18.3)	70.0 (58.3–85.0)	68.9 (19.8)	70.0 (55.5–80.5)^*^	65.8 (18.9)
Education high (*n =* 80)	71.0 (50.0–89.0)	64.6 (25.7)	77.5 (65.3–90.0)	75.4 (20.0)	73.0 (42.5–87.5)^*^	64.1 (27.4)
**Memory problems (*****n =*** **584)**	72.0 (60.0–84.8)	70.1 (20.2)	62.0 (50.0–80.0)	61.8 (23.0)	60.0 (39.8–80.0)	57.2 (25.2)
Education low (*n =* 187)	79.0 (60.0–81.0)	69.4 (20.7)	70.0 (50.0–81.0)	62.8 (26.1)	50.0 (33.0–72.0)^*^	51.0 (24.4)^*^
Education middle (*n =* 264)	71.0 (60.0–83.5)	69.5 (20.3)	61.0 (29.8–80.3)	62.0 (19.6)	67.5 (39.5–80.0)^*^	58.5 (26.4)^*^
Education high (*n =* 133)	73.5 (61.5–91.3)	73.1 (19.8)	67.0 (40.0–79.0)	58.8 (24.7)	68.0 (40.8–82.0)^*^	61.8 (23.4)^*^
**Depression or anxiety disorder (*****n =*** **1,903)**	70.0 (52.0–80.0)	66.6 (19.5)	66.0 (50.0–80.0)	63.6 (19.0)	60.0 (40.0–78.0)	58.2 (23.1)
Education low (*n =* 497)	72.0 (61.0–83.0)^*^	70.9 (17.9)^*^	65.0 (49.8–79.0)	61.9 (21.1)	50.0 (38.0–71.0)^*^	52.0 (24.3)^*^
Education middle (*n =* 918)	70.0 (50.0–80.0)^*^	64.9 (20.6)^*^	65.0 (50.0–80.0)	63.2 (18.9)	60.0 (40.0–78.0)^*^	58.1 (23.6)^*^
Education high (*n =* 488)	69.5 (52.0–80.0)^*^	65.6 (16.7)^*^	70.0 (60.0–80.0)	67.6 (15.1)	68.0 (50.0–80.0)^*^	62.9 (20.3)^*^
**Other chronic health conditions (*****n =*** **1,084)**	72.0 (60.0–85.0)	69.0 (20.8)	70.0 (56.5–80.0)	66.7 (19.5)	60.0 (39.0–72.0)	55.0 (23.7)
Education low (*n =* 313)	64.0 (50.0–83.0)	64.4 (22.8)	70.0 (50.0–80.0)	65.1 (19.7)	57.0 (32.5–74.5)	53.6 (24.7)
Education middle (*n =* 518)	75.5 (62.0–81.8)	71.5 (18.4)	70.0 (58.0–80.0)	66.3 (19.5)	55.0 (31.0–71.0)	52.4 (24.4)
Education high (*n =* 253)	80.0 (60.0–86.8)	69.6 (23.8)	73.0 (60.0–81.0)	69.6 (19.1)	61.0 (40.0–76.8)	59.3 (21.5)
**No chronic health conditions (*****n =*** **5,046)**	85.0 (79.0–91.0)	83.2 (13.9)	85.0 (79.0–92.0)	83.8 (13.6)	83.0 (72.0–91.0)	81.0 (15.7)
Education low (*n =* 1,160)	85.0 (71.0–92.0)	81.6 (16.2)^*^	89.0 (79.0–95.0)	84.0 (14.7)	82.0 (70.3–92.0)	79.9 (18.5)
Education middle (*n =* 2,583)	85.0 (80.0–91.0)	83.6 (13.0)^*^	86.5 (80.0–93.0)	84.5 (12.5)	82.0 (72.0–91.0)	81.0 (15.8)
Education high (*n =* 1,326)	85.0 (80.0–93.0)	84.7 (11.2)^*^	85.0 (78.3–90.0)	82.5 (14.5)	84.0 (75.0–90.0)	81.5 (14.0)

* *Statistically significantly different between subgroups based on level of education; p < 0.05*.

### Factors Associated With EQ-5D-5L Transformed Level Sum Score and EQ VAS

In Table 4, the univariate regression outcomes for the tLSS and EQ VAS are presented for each country separately. Negative coefficients represent a worse outcome compared to the reference group. Compared to the respondents with a high level of education, low and middle levels of education were associated with a worse outcome for both the tLSS and EQ VAS in the UK and Dutch samples, but not in the Italian sample. Low and middle levels of education (compared to a high level) were associated with a better outcome based on the tLSS for the Italian sample; however, no significant relation was seen based on the EQ VAS. Having an increased number of chronic health conditions was consistently associated with a decreased health outcome. Other factors associated with the outcomes are presented in Table 4.

**Table 4:** Univariate analyses of participant characteristics and the EQ-5D-5L transformed level sum score and EQ VAS in the three countries.

	EQ-5D-5L level sum score^*^						**EQ VAS**					
**Characteristic**	**Italy**		**Netherlands**		**UK**		**Italy**		**Netherlands**		**UK**	
	**Coef**.	***P*** **-value**	**Coef.**	***P*** **-value**	**Coef.**	***P*** **-value**	**Coef.**	***P*** **-value**	**Coef.**	***P-*value**	**Coef.**	***P*** **-value**
**Sex**		**<0.001**		**<0.001**		**0.005**		**<0.001**		**<0.001**		**0.064**
Male	2.160		2.595		1.667		2.287		3.467		1.245	
**Female (ref)**												
**Age category**		**<0.001**		**0.112**		**<0.001**		**<0.001**		**<0.001**		**<0.001**
18– <25 year	2.984		1.375		4.919		4.090		4.353		3.394	
25– <40 year	0.901		1.419		3.982		1.930		1.064			
40– <60 year (ref)											3.894	
60–75 year	−0.935		0.220		1.484		−2.318		1.584		2.921	
**Highest level of education**		**0.038**		**<0.001**		**<0.001**		**0.095**		**<0.001**		**<0.001**
Low	1.368		−5.486		−6.325		−0.242		−3.342		−6.781	
Middle	1.760		−1.774		−2.602		1.216		−0.296		−2.294	
**High (ref)**												
**Work status**		**<0.001**		**<0.001**		**<0.001**		**<0.001**		**<0.001**		**<0.001**
**Employed (ref)**												
Unemployed	−0.957		−6.180		−9.026		−1.342		−4.574		−8.249	
Looking after others	−0.690		−5.683		−1.940		−1.941		−4.566		−1.305	
Student	1.834		−0.783		0.678		2.347		−0.608		−0.702	
Retired	−2.406		−3.013		−5.558		−3.568		−1.439		−2.966	
Unable to work	−24.509		−20.163		−36.006		−31.010		−20.512		−29.239	
**Household income**		**<0.001**		**<0.001**		**<0.001**		**0.009**		**<0.001**		**<0.001**
Low	0.104		−6.239		−11.122		−0.338		−8.328		−10.016	
Middle	1.372		−1.633		−5.141		0.916		−1.63		−5.097	
**High (ref)**												
Do not know/do not want to tell	2.758		0.263		−4.762		2.826		−0.945		−3.752	
**Number of chronic health conditions**		**<0.001**		**<0.001**		**<0.001**		**<0.001**		**<0.001**		**<0.001**
**No condition (ref)**												
1 condition	−8.023		−10.348		−11.894		−9.780		−10.083		−11.649	
2 conditions	−13.974		−17.307		−23.462		−14.965		−17.908		−22.196	
3 conditions	−16.204		−24.081		−34.620		−17.974		−22.625		−27.615	
4 conditions	−26.198		−29.456		−44.110		−25.209		−28.014		−38.379	
≥5 conditions	−25.573		−34.885		−50.660		−25.461		−32.805		−41.759	

* *Transformed EQ-5D-5L level sum score*.

Results of the multivariable analyses are presented in Table 5. Sex and age were used in the first step and were significantly associated with the outcomes studied, with exceptions being age in the Dutch sample for the tLSS and sex in the UK sample for the EQ VAS. However, the effect of sex disappeared in all the samples, except for the Italian sample for the tLSS, when the level of education, work status, household income, and a number of chronic health conditions were included in the second step. The effect of age disappeared in the Italian sample for both outcomes studied.

**Table 5:** Multivariable analyses of participant characteristics and the EQ-5D-5L transformed level sum score and EQ VAS in the three countries.

	EQ-5D-5L level sum score^*^						**EQ VAS**					
**Characteristic**	**Italy**		**Netherlands**		**UK**		**Italy**		**Netherlands**		**UK**	
	**Coef.**	***P*** **-value**	**Coef.**	***P*** **-value**	**Coef.**	***P*** **-value**	**Coef.**	***P*** **-value**	**Coef.**	***P*** **-value**	**Coef.**	***P*** **-value**
Intercept	93.390	**<0.001**	96.488	**<0.001**	96.984	**<0.001**	81.741	**<0.001**	83.561	**<0.001**	81.988	**<0.001**
**Sex**		**0.002**		0.907		0.098		0.073		0.218		
Male	1.142		−0.050		−0.680		1.078		0.723			
**Female (ref)**												
**Age category**		0.316				**0.007**		0.132		**0.004**		**0.011**
18– <25 year	0.990				0.512		2.157		1.168		0.190	
25– <40 year	−0.251				0.218		0.728		−1.410		0.724	
40– <60 year (ref)												
60–75 year	0.683				2.753		−1.396		2.334		3.697	
**Highest level of education**		0.679		0.180		**<0.001**				0.072		**0.017**
Low	0.515		−0.916		−0.261				1.197		−2.015	
Middle	0.497		0.024		0.088				1.638		−0.061	
**High (ref)**												
**Work status**		**<0.001**		**<0.001**		**<0.001**		**<0.001**		**<0.001**		**<0.001**
**Employed (ref)**												
Unemployed	−0.459		−3.853		−5.051		−0.835		−2.170		−4.305	
Looking after others	−0.407		−2.512		−0.954		−1.378		−1.289		−0.203	
Student	0.409		−1.861		−1.366		−0.124		−1.765		−2.327	
Retired	−0.415		0.360		−4.258		0.621		−0.843		−2.271	
Unable to work	−17.344		−10.234		−22.548		−23.249		−10.856		−16.990	
**Household income**		**0.003**		0.402		0.263		0.159		**0.005**		0.069
Low	0.786		−0.059		−0.772		0.364		−3.042		−1.084	
Middle	1.518		0.412		−0.995		1.005		−0.148		−1.637	
**High (ref)**												
Do not know/do not want to tell	2.131		0.947		−0.381		2.062		−0.761		0.229	
**Number of chronic health conditions**		**<0.001**		**<0.001**		**<0.001**		**<0.001**		**<0.001**		**<0.001**
**No disease (ref)**												
1 disease	−7.670		−9.053		−9.851		−9.157		−8.763		−10.006	
2 diseases	−13.337		−14.554		−18.762		−13.889		−15.210		−18.555	
3 diseases	−15.300		−20.144		−26.676		−16.524		−18.561		−21.590	
4 diseases	−25.079		−24.514		−35.618		−23.563		−23.255		−31.936	
≥5 diseases	−23.031		−29.564		−40.170		−22.084		−27.558		−33.595	
*F*-value	73.18	**<0.001**	122.34	**<0.001**	256.00	**<0.001**	38.53	**<0.001**	55.78	**<0.001**	100.24	**<0.001**
*R*-squared	0.316		0.394		0.543		0.179		0.261		0.306	

*Bold values are statistically significant values*.

* *Transformed EQ-5D-5L level sum score*.

After controlling for other factors, a low level of education was associated with a worse tLSS and worse EQ VAS in the UK sample compared to the respondents with a high level of education. In the different country samples, having an increased number of chronic health conditions was consistently associated with a lower health outcome. Inability to work proved to be another factor that had a high impact in all the samples and on both the outcomes. The explained variability (*R*^2^) of the models was higher for the models based on the tLSS (31.6–54.3%) compared to the EQ VAS (17.9–30.6%), and highest for the UK and lowest for Italy.

Multivariable analyses were also conducted in the subgroups of participants with and without chronic health complaints, separately for each country (Appendices 2A,B). Inability to work and an increased number of chronic conditions were especially associated with a worse tLSS and worse EQ VAS in the samples from all the countries. The explained variability of the models varied largely from 9.1% for the EQ VAS in the Italian sample to 44.6% for the tLSS in the UK sample. Work status and, to a lower extent, household income were associated with the outcomes in the subgroup without any chronic health complaint. The explained variability of the models was very low (1.2–4.2%).

## Discussion

This study explored the potential of the EQ-5D-5L LSS as an outcome measure in the health inequality analyses relative to the EQ VAS. As hypothesized, our study showed that the EQ-5D-5L LSS tended to better discriminate between participants with a different level of education compared to the EQ VAS, both in the general sample as well as in subgroups of participants with a specific chronic health condition. Furthermore, the present study showed that the health outcomes differed among the three countries studied, with the worst outcomes for participants from the UK and best outcomes for participants from Italy. Participants with a low level of education had worst outcomes and those with a high level of education had the best outcomes in both the UK and the Netherlands, with larger differences among subgroups in the UK. Health inequalities were thus found to be highest in the UK and smallest in Italy, confirming our hypothesis. Multivariable analyses showed generally stronger predictive relations in the UK and with the EQ-5D-5L LSS. The presence of chronic health conditions and being unable to work were strong independent predictors, canceling out the educational effects (apart from a small effect in the UK).

Results from our study are in line with earlier studies that showed that the EQ-5D reflects health inequalities: low SES (defined as low educational level/low status/unskilled manual group/low income/low social class) is associated with a lower EQ outcome (25, 34–40). Some studies used the EQ VAS as an outcome (25, 37, 38), while some used the separate EQ-5D domains as an outcome (25, 34, 37–39), and some others used the EQ utility score as an outcome (35, 36, 39, 40). Use of the separate domains might not be very useful as to disperse populations by the level of inequality, health differences are often summarized into one single value (2, 7, 10, 16). An earlier study used both the EQ-5D utility score and EQ VAS as outcomes and showed that the EQ-5D utility score better discriminated between ethnicity groups than the EQ VAS (41), which is in line with our results. However, in our study, in the UK participants with memory problems and in the subgroups of participants without a chronic health condition, the EQ VAS was discriminative between participants with a different level of education whereas the EQ-5D was not. Potential causes could be the mediation effect of the inability to work and the presence of chronic health conditions, and/or the fact that EQ VAS and EQ-5D scores could assess slightly different outcomes, which was also shown in previous work (42). It was reported that EQ VAS scores could be predicted from EQ-5D scores, but there was the contribution from other factors also, including psychological status, age, education, and distress (42). The EQ-5D-5L LSS thus seems to better discriminate between subgroups, which is valuable in health inequality analysis. However, the use of an instrument that includes more than one question also includes some disadvantages, namely the extra time needed to complete the instrument and the corresponding higher burden for those completing it, which might result in a potentially lower response rate. It is important to consider whether the improved discriminative power outweighs these disadvantages.

Comparison of our findings on differences among countries are in line with the existing literature and confirm our hypothesis (37). A large European comparison study found that the relative index of inequality was the highest in the UK, lower in the Netherlands, and the lowest in Italy, when these three countries were compared (7). The same pattern was seen in a study on socioeconomic inequalities in self-assessed health in 17 European countries (43). Suggested potential factors that might contribute to these smaller health inequalities in Italy include the Mediterranean diet and the relatively low number of female smokers (44, 45). Within our Italian sample, it was surprising that differences in SES did not seem to exist. Previous studies reported the same patterns of worse health outcomes being associated with a low level of education and better health outcomes with a high level of education (7, 43).

Our results indicate that having a chronic health condition does not explain all the differences that arise from the level of education. This confirms earlier studies that showed that a low SES is associated with worse health outcomes, even after controlling for having chronic diseases (25, 41, 46, 47), although the inability to work (the companion variable explaining the education effect) was not always identically covered. Furthermore, within samples with a specific disease, studies also reported that a low level of education is associated with worse outcomes (40, 48–51). However, our results were inconsistent after controlling for relevant demographic factors. Apart from an association between low level of education and worse EQ-5D-5L LSS in the Dutch sample and low level of education and worse EQ VAS in the UK sample, no other association between educational level and outcomes was observed. Thus, other factors seemed to be stronger associated with a worse health outcome, with a consistently strong negative impact of having a chronic health condition. This association was shown to increase with an increase in the number of chronic health conditions. In the subgroup analysis, it is noteworthy that for the UK, low and middle income had a borderline significant negative impact on tLSS for the diseased subgroup, which may be indicative of more pronounced income inequality effects on health in the UK. The subgroup analysis also showed how unable to work had a much larger impact on tLSS in the diseased group when compared to the healthy subgroup for all countries, as expected.

The present study included some strengths and limitations. Strengths included the large sample size and the invitation of persons from three countries that were representative of their population with respect to age, sex, and educational level. Also, some limitations should be considered. By the use of a web-based survey, we only included participants who had access to a computer and internet and who were able to read and understand the survey, which may have led to participation bias as persons with low health literacy (often those with a low SES) may not have been included. We tried to mitigate this by selecting participants with certain characteristics (e.g., age, sex, and educational level) from the existing large panel in order to increase the representativeness of the study sample for the adult population in the selected countries. Also, a detailed analysis of non-responders was not possible due to the system of recruitment used.

Second, we used the educational level as a proxy for SES rather than an indicator that also incorporates income level or financial security. However, educational level is a common and widely used proxy for SES in the field of social epidemiology. It is a characteristic that has strong associations with a multitude of factors linked to health such as risky health behavior, limited access to financial and social resources, and type of work and residency (52–54).

Third, the outcome measure in our study was the LSS rather than the utility scores calculated with value sets of the three countries. Value sets for the EQ-5D reflect the preferences of the population of a country for the EQ-5D-5L health states, and the utility scores for the same health state calculated with different value sets vary substantially (55). Currently, there is no EQ-5D-5L value set available for Italy. Therefore, in order to use the EQ-5D-5L utility scores as an outcome measure, a proxy value set should be applied to assess the EQ-5D-5L utilities for the Italian study sample. In our study, the choice of the alternative value to assess utility scores for the Italian respondents, the Dutch or the UK value set, is arbitrary. The use of one of these value sets would therefore mean that differences in the utility scores between the Italian and UK or Dutch respondents reflect the differences in the EQ-5D-5L profile only and not the differences in the preferences for health states between these countries.

## Conclusion

The present study showed that in three different European countries, EQ-5D measures show the presence of education-dependent health inequalities, which are universally explained in regression analysis by, independently, the presence of chronic health conditions and the inability to work. In stratified analysis, the EQ-5D-5L LSS shows slightly better discrimination between participants with different levels of SES compared to the EQ VAS.

## Data Availability Statement

The data analyzed in this study is subject to the following licenses/restrictions: the datasets generated and/or analyzed during the current study are not publicly available due to privacy/ethical restrictions but are available from the corresponding author on reasonable request. Requests to access these datasets should be directed to Inge Spronk, i.spronk@erasmusmc.nl.

## Ethics Statement

The studies involving human participants were reviewed and approved by Leids Universitair Centrum—Commissie Medische Ethiek. The participants provided their written informed consent to participate in this study.

## Author Contributions

IS conceptualized and designed the study, analyzed and interpreted data, drafted the initial manuscript, and reviewed and revised the manuscript. SP, EL, and MJ conceptualized and designed the study, interpreted data, and reviewed and critically revised the manuscript. JH and GB conceptualized and designed the study, analyzed and interpreted data, and reviewed and critically revised the manuscript. All authors approved the final manuscript as submitted and agree to be accountable for all aspects of the work.

## Funding

This work was supported by the EuroQol Research Foundation (Grant No.: 20180630).

## Conflict of Interest

The authors declare that the research was conducted in the absence of any commercial or financial relationships that could be construed as a potential conflict of interest.

## Publisher's Note

All claims expressed in this article are solely those of the authors and do not necessarily represent those of their affiliated organizations, or those of the publisher, the editors and the reviewers. Any product that may be evaluated in this article, or claim that may be made by its manufacturer, is not guaranteed or endorsed by the publisher.

## References

[1] Marmot M. Social determinants of health inequalities. Lancet. (2005) 365:1099–104. 10.1093/acprof:oso/9780198565895.001.0001 15781105

[2] Mackenbach JP. Health Inequalities: Europe in Profile. Rotterdam: COI for the Department of Health. (2006).

[3] Graham H. Understanding Health Inequalities. Berkshire: McGraw-Hill Education. (2009).

[4] Phelan JC Link BG Tehranifar P. Social conditions as fundamental causes of health inequalities: theory, evidence, policy implications. J Health Soc Behav. (2010) 51:S28–40. 10.1177/0022146510383498 20943581

[5] World Health O. Handbook on Health Inequality Monitoring: With a Special Focus on Low-and Middle-Income Countries. Geneva: World Health Organization. (2013).

[6] Woodward A Kawachi I. Why reduce health inequalities? J Epidemiol Commun Health. (2000) 54:923–9. 10.1136/jech.54.12.923 11076989 PMC1731601

[7] Mackenbach JP Stirbu I Roskam AJR Schaap MM Menvielle G. Socioeconomic inequalities in health in 22 European countries. N Engl J Med. (2008) 358:2468–81. 10.1056/NEJMsa0707519 18525043

[8] Oliver S Kavanagh J Caird J Lorenc T Oliver K Harden A. Health Promotion, Inequalities and Young People's Health: A Systematic Review of Research London. (2008).

[9] Arcaya MC Arcaya AL Subramanian S. Inequalities in health: definitions, concepts, and theories. Glob Health Action. (2015) 8:27106. 10.3402/gha.v8.27106 26112142 PMC4481045

[10] Kjellsson G Gerdtham UG Petrie D. Lies, damned lies, and health inequality measurements: understanding the value judgments. Epidemiology. (2015) 26:673. 10.1097/EDE.0000000000000319 26133019 PMC4521896

[11] Mackenbach JP. The persistence of health inequalities in modern welfare states: the explanation of a paradox. Soc Sci Med. (2012) 75:761–9. 10.1016/j.socscimed.2012.02.031 22475407

[12] Hatzenbuehler ML Phelan JC Link BG. Stigma as a fundamental cause of population health inequalities. Am J Public Health. (2013) 103:813–21. 10.2105/AJPH.2012.301069 23488505 PMC3682466

[13] Garcia SF Cella D Clauser SB Flynn KE Lad T Lai JS. Standardizing patient-reported outcomes assessment in cancer clinical trials: a patient-reported outcomes measurement information system initiative. J Clin Oncol. (2007) 25:5106–12. 10.1200/JCO.2007.12.2341 17991929

[14] Cella D Riley W Stone A Rothrock N Reeve B Yount S. The Patient-Reported Outcomes Measurement Information System (PROMIS) developed and tested its first wave of adult self-reported health outcome item banks: 2005-2008. J Clin Epidemiol. (2010) 63:1179–94. 10.1016/j.jclinepi.2010.04.011 20685078 PMC2965562

[15] Deshpande PR Rajan S Sudeepthi BL Nazir CA. Patient-reported outcomes: a new era in clinical research. Perspect Clin Res. (2011) 2:137. 10.4103/2229-3485.86879 22145124 PMC3227331

[16] Van Doorslaer E Gerdtham UG. Does inequality in self-assessed health predict inequality in survival by income? Evidence from Swedish data. Soc Sci Med. (2003) 57:1621–9. 10.1016/S0277-9536(02)00559-2 12948571

[17] Gardner DG Cummings LL Dunham RB Pierce JL. Single-item versus multiple-item measurement scales: An empirical comparison. Educ Psychol Meas. (1998) 58:898–915. 10.1177/0013164498058006003

[18] Gogol K Brunner M Goetz T Martin R Ugen S Keller U. “My questionnaire is too long!” The assessments of motivational-affective constructs with three-item and single-item measures. Contemp Educ Psychol. (2014) 39:188–205. 10.1016/j.cedpsych.2014.04.002

[19] Golicki D Niewada M Karlińska A Buczek J Kobayashi A Janssen M. Comparing responsiveness of the EQ-5D-5L, EQ-5D-3L and EQ VAS in stroke patients. Qual Life Res. (2015) 24:1555–63. 10.1007/s11136-014-0873-7 25425288 PMC4457098

[20] Roelen CA Heymans MW Twisk JW Laaksonen M Pallesen S Magerøy N. Health measures in prediction models for high sickness absence: single-item self-rated health versus multi-item SF-12. Eur J Public Health. (2015) 25:668–72. 10.1093/eurpub/cku192 25465915

[21] Diamantopoulos A Sarstedt M Fuchs C Wilczynski P Kaiser S. Guidelines for choosing between multi-item and single-item scales for construct measurement: a predictive validity perspective. J Acad Market Sci. (2012) 40:434–49. 10.1007/s11747-011-0300-3

[22] Brooks R. EuroQol: the current state of play. Health Policy. (1996) 37:53–72. 10.1016/0168-8510(96)00822-6 10158943

[23] Gundgaard J Lauridsen J. A decomposition of income-related health inequality applied to EQ-5D. Eur J Health Econ. (2006) 7:231–7. 10.1007/s10198-006-0360-3 16763803

[24] Swinburn P Lloyd A Boye K Edson-Heredia E Bowman L Janssen B. Development of a disease-specific version of the EQ-5D-5L for use in patients suffering from psoriasis: lessons learned from a feasibility study in the UK. Value Health. (2013) 16:1156–62. 10.1016/j.jval.2013.10.003 24326169

[25] Mielck A Vogelmann M Leidl R. Health-related quality of life and socioeconomic status: inequalities among adults with a chronic disease. Health Qual Life Outcomes. (2014) 12:1–10. 10.1186/1477-7525-12-58 24761773 PMC4011770

[26] EuroQol Research Foundation. About the EQ-5D-3L. (2019). Available online at: https://euroqol.org/eq-5d-instruments/eq-5d-3l-about/ (accessed April 20, 2019).

[27] Herdman M Gudex C Lloyd A Janssen M Kind P Parkin D. Development and preliminary testing of the new five-level version of EQ-5D (EQ-5D-5L). Qual Life Res. (2011) 20:1727–36. 10.1007/s11136-011-9903-x 21479777 PMC3220807

[28] Eichler HG Kong SX Gerth WC Mavros P Jönsson B. Use of cost-effectiveness analysis in health-care resource allocation decision-making: how are cost-effectiveness thresholds expected to emerge? Value Health. (2004) 7:518–28. 10.1111/j.1524-4733.2004.75003.x 15367247

[29] Rios-Diaz AJ Lam J Ramos MS Moscoso AV Vaughn P Zogg CK. Global patterns of QALY and DALY use in surgical cost-utility analyses: a systematic review. PLoS ONE. (2016) 11:e0148304. 10.1371/journal.pone.0148304 26862894 PMC4749322

[30] Voormolen DC Cnossen MC Polinder S Gravesteijn BY Von Steinbuechel N Real RGL. Prevalence of post-concussion-like symptoms in the general population in Italy, The Netherlands and the UK. Brain Injury. (2019) 201:1–9. 10.1080/02699052.2019.1607557 31032649

[31] Rabin R Charro FD. EQ-5D: a measure of health status from the EuroQol Group. Ann Med. (2001) 33:337–43. 10.3109/07853890109002087 11491192

[32] Fylkesnes K Jakobsen MD Henriksen NO. The value of general health perception in health equity research: A community-based cohort study of long-term mortality risk (Finnmark cohort study 1987-2017). SSM Popul Health. (2021) 15:100848. 10.1016/j.ssmph.2021.100848 34195347 PMC8237603

[33] Janssen MF Bonsel GJ Luo N. Is EQ-5D-5L better than EQ-5D-3L? A head-to-head comparison of descriptive systems and value sets from seven countries. Pharmacoeconomics. (2018) 36:675–97. 10.1007/s40273-018-0623-8 29470821 PMC5954015

[34] Kind P Dolan P Gudex C Williams A. Variations in population health status: results from a UK national questionnaire survey. BMJ. (1998) 316:736–41. 10.1136/bmj.316.7133.736 9529408 PMC28477

[35] Burström K Johannesson M Diderichsen F. Swedish population health-related quality of life results using the EQ-5D. Qual Life Res. (2001) 10:621–35. 10.1023/A:1013171831202 11822795

[36] Sullivan PW Ghushchyan V. Preference-based EQ-5D index scores for chronic conditions in the United States. Med Decision Making. (2006) 26:410–20. 10.1177/0272989X06290495 16855129 PMC2634296

[37] König HH Heider D Lehnert T Riedel-Heller SG Angermeyer MC Matschinger H. Health status of the advanced elderly in six European countries: results from a representative survey using EQ-5D and SF-12. Health Qual Life Outcomes. (2010) 8:143. 10.1186/1477-7525-8-143 21114833 PMC3009699

[38] Li H Wei X Ma A Chung RY. Inequalities in health status among rural residents: EQ-5D findings from household survey China. Int J Equity Health. (2014) 13:41. 10.1186/1475-9276-13-41 24885378 PMC4030034

[39] Zhou Z Fang Y Zhou Z Li D Wang D Li Y. Assessing income-related health inequality and horizontal inequity in China. Soc Indic Res. (2017) 132:241–56. 10.1007/s11205-015-1221-1

[40] Arrospide A Machón M Ramos-Goñi JM Ibarrondo O Mar J. Inequalities in health-related quality of life according to age, gender, educational level, social class, body mass index and chronic diseases using the Spanish value set for Euroquol 5D-5L questionnaire. Health Qual Life Outcomes. (2019) 17:69. 10.1186/s12955-019-1134-9 30999899 PMC6472013

[41] Lubetkin EI Jia H Franks P Gold MR. Relationship among sociodemographic factors, clinical conditions, and health-related quality of life: examining the EQ-5D in the US general population. Qual Life Res. (2005) 14:2187–96. 10.1007/s11136-005-8028-5 16328899

[42] Whynes DK Health TGJ. Correspondence between EQ-5D health state classifications and EQ VAS scores. Health Qual Life Outcomes. (2008) 6:94. 10.1186/1477-7525-6-94 18992139 PMC2588564

[43] Hu Y van Lenthe FJ Borsboom GJ Looman CW Bopp M Burström B. Trends in socioeconomic inequalities in self-assessed health in 17 European countries between 1990 and 2010. J Epidemiol Community Health. (2016) 70:644–52. 10.1136/jech-2015-206780 26787202

[44] Mackenbach JP Cavelaars A Kunst AE Groenhof F. Socioeconomic inequalities in cardiovascular disease mortality. An international study. Eur Heart J. (2000) 21:1141–51. 10.1053/euhj.1999.1990 10924297

[45] Knoops KT de Groot LC Kromhout D Perrin AE Moreiras-Varela O. Mediterranean diet, lifestyle factors, and 10-year mortality in elderly European men and women: the HALE project. JAMA. (2004) 292:1433–9. 10.1001/jama.292.12.1433 15383513

[46] Ko Y Coons SJ. Self-reported chronic conditions and EQ-5D index scores in the US adult population. Curr Med Res Opin. (2006) 22:2065–71. 10.1185/030079906X132622 17031907

[47] Mielck A Reitmeir P Vogelmann M Leidl R. Impact of educational level on health-related quality of life (HRQL): results from Germany based on the EuroQol 5D (EQ-5D). Eur J Public Health. (2013) 23:45–9. 10.1093/eurpub/ckr206 22434205

[48] Wexler D Grant R Wittenberg E Bosch J Cagliero E Delahanty L. Correlates of health-related quality of life in type 2 diabetes. Diabetologia. (2006) 49:1489–97. 10.1007/s00125-006-0249-9 16752167

[49] Schweikert B Hunger M Meisinger C König HH Gapp O Holle R. Quality of life several years after myocardial infarction: comparing the MONICA/KORA registry to the general population. Eur Heart J. (2009) 30:436–43. 10.1093/eurheartj/ehn509 19019995

[50] Stafford M Soljak M Pledge V Mindell J. Socio-economic differences in the health-related quality of life impact of cardiovascular conditions. Eur J Public Health. (2012) 22:301–5. 10.1093/eurpub/ckr007 21398378 PMC3358629

[51] Xu RH Cheung AWL Wong ELY. Examining the health-related quality of life using EQ-5D-5L in patients with four kinds of chronic diseases from specialist outpatient clinics in Hong Kong SAR China. Patient Prefer Adherence. (2017) 11:1565. 10.2147/PPA.S143944 28979104 PMC5602472

[52] Ross CE Wu CL. The links between education and health. Am Sociol Rev. (1995) 60:719–45. 10.2307/2096319

[53] Daly MC Duncan GJ McDonough P Williams DR. Optimal indicators of socioeconomic status for health research. Am J Public Health. (2002) 92:1151–7. 10.2105/AJPH.92.7.1151 12084700 PMC1447206

[54] Lahelma E Martikainen P Laaksonen M Aittomaki A. Pathways between socioeconomic determinants of health. J Epidemiol Community Health. (2004) 58:327–32. 10.1136/jech.2003.011148 15026449 PMC1732713

[55] Gerlinger C Bamber L Leverkus F Schwenke C Haberland C Schmidt G. Comparing the EQ-5D-5L utility index based on value sets of different countries: impact on the interpretation of clinical study results. BMC Res Notes. (2019) 12:18. 10.1186/s13104-019-4067-9 30642397 PMC6332559

