# Supplementary material for: Health Inequality Analysis in Europe: Exploring the Potential of the EQ-5D as Outcome
Source: Front Public Health. 2021 Nov 4;9:744405. doi: 10.3389/fpubh.2021.744405 (PMC8599146; doi:10.3389/fpubh.2021.744405)
Supplement: Supplementary file 1 [file Table_1.DOCX]

**Appendix 1a. Multivariable analyses of participant characteristics and the EQ-5D-5L transformed level sum score and EQ VAS in the three countries for participants with a chronic health complaint**

|  | **EQ-5D-5L Level Sum Score*** | | | | | |  | **EQ VAS** | | | | | |
| --- | --- | --- | --- | --- | --- | --- | --- | --- | --- | --- | --- | --- | --- |
| **Characteristic** | **Italy** | | **Netherlands** | | **United Kingdom** | |  | **Italy** | | **Netherlands** | | **United Kingdom** | |
|  | **Coef.** | **P value** | **Coef.** | **P value** | **Coef.** | **P value** |  | **Coef.** | **P value** | **Coef.** | **P value** | **Coef.** | **P value** |
| **Intercept** | 85.282 | **<0.001** | 87.883 | **<0.001** | 88.777 | **<0.001** |  | 72.000 | **<0.001** | 75.454 | **<0.001** | 72.316 | **<0.001** |
|  |  |  |  |  |  |  |  |  |  |  |  |  |  |
| **Sex** |  | 0.111 |  | 0.872 |  | 0.080 |  |  | 0.361 |  | 0.168 |  |  |
| Male | 1.161 |  | -0.120 |  | -1.185 |  |  | 0.945 |  | 1.263 |  |  |  |
| Female (ref) |  |  |  |  |  |  |  |  |  |  |  |  |  |
|  |  |  |  |  |  |  |  |  |  |  |  |  |  |
| **Age category** |  | **0.042** |  |  |  | **<0.001** |  |  | 0.255 |  | **0.016** |  | **0.004** |
| 18 - <25yr | 0.394 |  |  |  | 1.792 |  |  | 4.536 |  | 1.498 |  | -0.087 |  |
| 25 - <40yr | -1.517 |  |  |  | 0.065 |  |  | 0.812 |  | -2.292 |  | 0.376 |  |
| 40 - <60yr (ref) |  |  |  |  |  |  |  |  |  |  |  |  |  |
| 60 - 75yr | 2.332 |  |  |  | 5.089 |  |  | -0.796 |  | 2.557 |  | 5.696 |  |
|  |  |  |  |  |  |  |  |  |  |  |  |  |  |
| **Highest level of education** |  | 0.494 |  | 0.158 |  | 0.696 |  |  |  |  | 0.648 |  | **0.015** |
| Low | 1.462 |  | -1.757 |  | -0.074 |  |  |  |  | 0.432 |  | -3.103 |  |
| Middle | 0.855 |  | -0.278 |  | 0.536 |  |  |  |  | 1.037 |  | -0.217 |  |
| High (ref) |  |  |  |  |  |  |  |  |  |  |  |  |  |
|  |  |  |  |  |  |  |  |  |  |  |  |  |  |
| **Work status** |  | **<0.001** |  | **<0.001** |  | **<0.001** |  |  | **<0.001** |  | **<0.001** |  | **<0.001** |
| Employed (ref) |  |  |  |  |  |  |  |  |  |  |  |  |  |
| Unemployed | -1.098 |  | -5.160 |  | -7.337 |  |  | -1.044 |  | -2.627 |  | -4.697 |  |
| Looking after others | 0.935 |  | -3.325 |  | -2.487 |  |  | -0.117 |  | -1.491 |  | -1.423 |  |
| Student | 2.303 |  | -3.978 |  | -3.027 |  |  | 0.759 |  | -2.559 |  | -2.385 |  |
| Retired | -1.662 |  | -0.390 |  | -7.269 |  |  | 1.384 |  | -1.857 |  | -3.971 |  |
| Unable to work | -18.477 |  | -10.922 |  | -24.534 |  |  | -22.929 |  | -10.912 |  | -17.821 |  |
|  |  |  |  |  |  |  |  |  |  |  |  |  |  |
| **Household income** |  | 0.098 |  | 0.296 |  | 0.055 |  |  | 0.593 |  | 0.179 |  | 0.153 |
| Low | 0.698 |  | 0.628 |  | -2.201 |  |  | 0.683 |  | -2.732 |  | -1.261 |  |
| Middle | 1.734 |  | 0.648 |  | -2.402 |  |  | 0.914 |  | -0.184 |  | -2.148 |  |
| High (ref) |  |  |  |  |  |  |  |  |  |  |  |  |  |
| Do not know/ do not want to tell | 2.778 |  | 2.233 |  | -2.258 |  |  | 2.442 |  | -1.008 |  | 0.434 |  |
|  |  |  |  |  |  |  |  |  |  |  |  |  |  |
| **Number of chronic health conditions** |  | **<0.001** |  | **<0.001** |  | **<0.001** |  |  | **<0.001** |  | **<0.001** |  | **<0.001** |
| 1 disease (ref) |  |  |  |  |  |  |  |  |  |  |  |  |  |
| 2 diseases | -5.708 |  | -5.434 |  | -8.624 |  |  | -4.808 |  | -6.406 |  | -8.477 |  |
| 3 diseases | -7.665 |  | -10.917 |  | -16.257 |  |  | -7.348 |  | -9.768 |  | -11.487 |  |
| 4 diseases | -17.313 |  | -15.278 |  | -25.457 |  |  | -14.514 |  | -14.590 |  | -22.005 |  |
| ≥5 diseases | -15.108 |  | -20.214 |  | -29.782 |  |  | -12.977 |  | -18.866 |  | -23.528 |  |
|  |  |  |  |  |  |  |  |  |  |  |  |  |  |
| **F value**  **R-squared** | 14.68  0.166 | **<0.001** | 30.27  0.227 | **<0.001** | 97.58  0.446 | **<0.001** |  | 8.26  0.091 | **<0.001** | 16.73  0.163 | **<0.001** | 38.53  0.225 | **<0.001** |

**Appendix 1b. Multivariable analyses of participant characteristics and the EQ-5D-5L transformed level sum score and EQ VAS in the three countries for participants without a chronic health complaint**

|  | **EQ-5D-5L Level Sum Score*** | | | | | |  | **EQ VAS** | | | | | |
| --- | --- | --- | --- | --- | --- | --- | --- | --- | --- | --- | --- | --- | --- |
| **Characteristic** | **Italy** | | **Netherlands** | | **United Kingdom** | |  | **Italy** | | **Netherlands** | | **United Kingdom** | |
|  | **Coef.** | **P value** | **Coef.** | **P value** | **Coef.** | **P value** |  | **Coef.** | **P value** | **Coef.** | **P value** | **Coef.** | **P value** |
| **Intercept** | 93.870 | **<0.001** | 96.211 | **<0.001** | 95.981 | **<0.001** |  | 82.114 | **<0.001** | 83.095 | **<0.001** | 81.911 | **<0.001** |
|  |  |  |  |  |  |  |  |  |  |  |  |  |  |
| **Sex** |  | **0.001** |  | 0.827 |  | 0.909 |  |  | 0.080 |  | 0.817 |  |  |
| Male | 1.147 |  | 0.089 |  | -0.046 |  |  | 1.234 |  | 0.169 |  |  |  |
| Female (ref) |  |  |  |  |  |  |  |  |  |  |  |  |  |
|  |  |  |  |  |  |  |  |  |  |  |  |  |  |
| **Age category** |  | **0.039** |  |  |  | 0.065 |  |  | 0.370 |  | 0.371 |  | 0.827 |
| 18 - <25yr | 1.296 |  |  |  | -1.753 |  |  | 0.753 |  | 1.076 |  | -0.087 |  |
| 25 - <40yr | 0.349 |  |  |  | -0.180 |  |  | 0.490 |  | -0.535 |  | 0.728 |  |
| 40 - <60yr (ref) |  |  |  |  |  |  |  |  |  |  |  |  |  |
| 60 - 75yr | -1.243 |  |  |  | -1.310 |  |  | -2.024 |  | 1.782 |  | 0.699 |  |
|  |  |  |  |  |  |  |  |  |  |  |  |  |  |
| **Highest level of education** |  | 0.672 |  | 0.925 |  | 0.483 |  |  |  |  | **0.028** |  | 0.670 |
| Low | -0.227 |  | -0.054 |  | -0.606 |  |  |  |  | 2.076 |  | -0.852 |  |
| Middle | 0.112 |  | 0.132 |  | -0.463 |  |  |  |  | 2.174 |  | -0.012 |  |
| High (ref) |  |  |  |  |  |  |  |  |  |  |  |  |  |
|  |  |  |  |  |  |  |  |  |  |  |  |  |  |
| **Work status** |  | **0.001** |  | **<0.001** |  | **0.016** |  |  | **0.006** |  | **<0.001** |  | **0.013** |
| Employed (ref) |  |  |  |  |  |  |  |  |  |  |  |  |  |
| Unemployed | 0.060 |  | -2.386 |  | -1.277 |  |  | -0.569 |  | -1.718 |  | -3.478 |  |
| Looking after others | -1.049 |  | -1.535 |  | 0.306 |  |  | -2.149 |  | -1.192 |  | 0.915 |  |
| Student | -0.491 |  | -0.298 |  | 0.497 |  |  | -0.468 |  | -1.237 |  | -2.193 |  |
| Retired | 0.806 |  | 1.094 |  | -0.087 |  |  | -0.234 |  | 0.714 |  | 0.125 |  |
| Unable to work | -12.253 |  | -8.689 |  | -5.695 |  |  | -23.954 |  | -12.553 |  | -9.303 |  |
|  |  |  |  |  |  |  |  |  |  |  |  |  |  |
| **Household income** |  | **0.018** |  | 0.442 |  | 0.400 |  |  | 0.264 |  | **0.036** |  | 0.475 |
| Low | 0.892 |  | -0.819 |  | 0.419 |  |  | 0.240 |  | -3.497 |  | -1.044 |  |
| Middle | 1.333 |  | 0.251 |  | 0.164 |  |  | 1.089 |  | -0.210 |  | -1.244 |  |
| High (ref) |  |  |  |  |  |  |  |  |  |  |  |  |  |
| Do not know/ do not want to tell | 1.602 |  | -0.185 |  | 1.179 |  |  | 1.931 |  | -0. 618 |  | -0.043 |  |
|  |  |  |  |  |  |  |  |  |  |  |  |  |  |
| **F value**  **R-squared** | 3.87  0.032 | **<0.001** | 5.81  0.042 | **<0.001** | 1.97  0.014 | **0.017** |  | 2.54  0.018 | **0.003** | 4.04  0.038 | **<0.001** | 1.84  0.012 | **0.033** |
