# Supplementary material for: Health Inequality Analysis in Europe: Exploring the Potential of the EQ-5D as Outcome
Source: Front Public Health. 2021 Nov 4;9:744405. doi: 10.3389/fpubh.2021.744405 (PMC8599146; doi:10.3389/fpubh.2021.744405)

## **Appendix 2A. Distribution of gender and age categories of the adult population of Italy (A), the Netherlands (B) and the United Kingdom (C)**

1. **Italy**


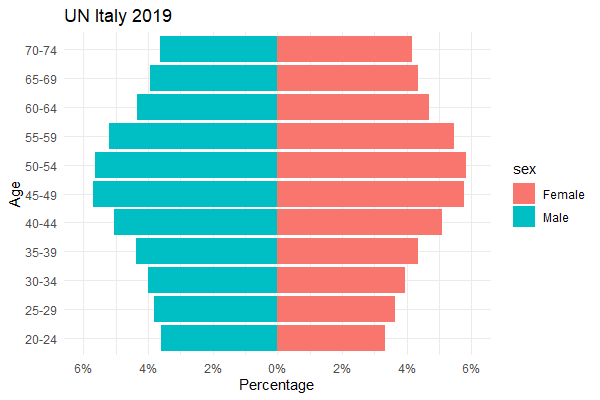


1. **The Netherlands**


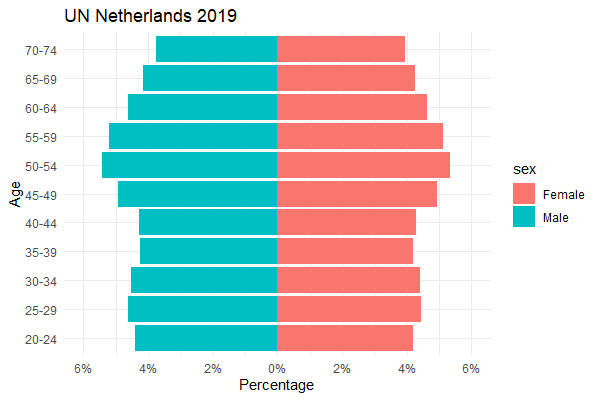


1. **United Kingdom (UK)**


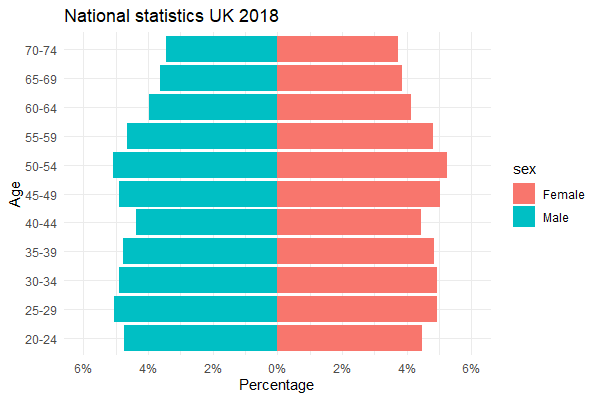


## **Appendix 2B. Distribution of educational level of the adult population of Italy (A), the Netherlands (B) and the United Kingdom (C)**

## **A. Italy**


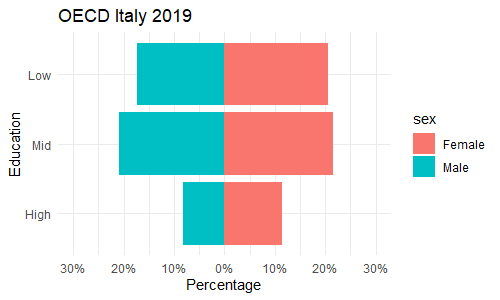


**B. The Netherlands**


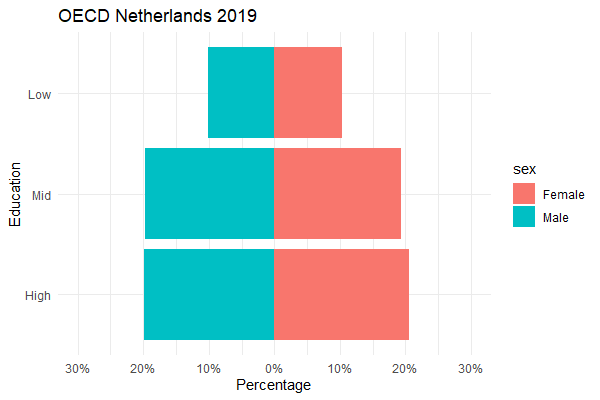


1. **United Kingdom (UK)**


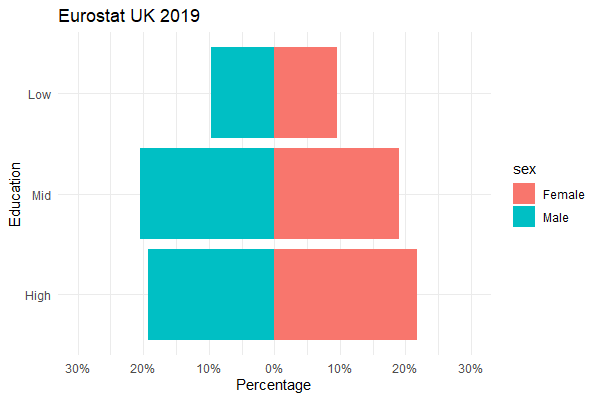

Supplement: Supplementary file 2 [file Table_2.docx]
